# Unicuspid Aortic Stenosis in a Patient with Turner Syndrome: A Case Report

**DOI:** 10.3389/fcvm.2014.00014

**Published:** 2014-12-23

**Authors:** Michael Essandoh, Karina Castellon-Larios, Alix Zuleta-Alarcon, Juan Guillermo Portillo, Juan A. Crestanello

**Affiliations:** ^1^Department of Anesthesiology, Wexner Medical Center, Ohio State University, Columbus, OH, USA; ^2^Division of Cardiothoracic Surgery, Department of Surgery, Wexner Medical Center, Ohio State University, Columbus, OH, USA

**Keywords:** Turner Syndrome, transesophageal echocardiography, unicuspid valve, aortic stenosis, electrocardiographically gated cardiac multidetector computed tomography

## Abstract

Congenital aortic valve anomalies are the cause of premature aortic stenosis in pediatric and younger adult populations. Despite being very rare, unicuspid aortic valves account for approximately 5% of isolated aortic valve replacements. Patients with aortic stenosis, present with the same symptomatology independent of leaflet morphology. However, the presence of bicuspid and unicuspid aortic stenosis is associated with a higher incidence of aortopathy, especially in Turner syndrome patients. Turner syndrome, an X monosomy, is associated with aortic valve anomalies, aortopathy, and hypertension. These risk factors lead to a higher incidence of aortic dissection in this population. Patients with Turner syndrome and aortic stenosis that present for aortic valve replacement should therefore undergo extensive aortic imaging prior to surgery. Transthoracic echocardiography is the diagnostic tool of choice for valvular pathology, yet it can misdiagnose unicuspid aortic valves as bicuspid valves due to certain similarities on imaging. Transesophageal echocardiography is a better tool for distinguishing between the two valvular abnormalities, although diagnostic errors can still occur. We present a case of a 50-year-old female with history of Turner syndrome and bicuspid aortic stenosis presenting for aortic valve replacement and ascending aorta replacement. Intraoperative transesophageal echocardiography revealed a stenotic unicommissural unicuspid aortic valve with an eccentric orifice, which was missed on preoperative imaging. This case highlights the importance of intraoperative transesophageal echocardiography in confirming preoperative findings, diagnosing further cardiac pathology, and ensuring adequate surgical repair.

## Introduction

Turner syndrome (TS), a monosomy of the X chromosome, is associated with multiple congenital cardiovascular abnormalities such as aortic valve (AV) anomalies and aortic coarctation. These conditions in addition to the presence of other risk factors such as underlying connective tissue disorder and hypertension lead to an accelerated rate of aortic dilatation and increased risk of aortic dissection in TS patients (1, 2). The presence of AV malformation leads to premature valvular stenosis from altered blood flow dynamics. In spite of the high incidence of AV anomalies in TS patients, the predominant pathology remains bicuspid aortic stenosis (AS) (1, 2). Congenital unicuspid aortic valve (UAV) is a very rare anomaly associated with premature AS and accounts for approximately 5% of all isolated stenotic AV replacements (3–5). Transthoracic echocardiography (TTE) fails to clearly define AV morphology in 10–40% of patients, despite being the primary imaging modality for the diagnosis and assessment of AS severity (3, 6, 7). We present a case of intraoperative diagnosis of unicuspid AS by transesophageal echocardiography (TEE) in a patient with TS presenting for aortic valve replacement (AVR) with a preoperative diagnosis of bicuspid AS.

## Case Report

A 50-year-old female with a complaint of exertional dyspnea, near-syncope, and angina was referred for cardiovascular evaluation at an outside facility. The patient’s past medical history was significant for TS, hypertension, hyperlipidemia, and diabetes mellitus type 2. TTE demonstrated normal left ventricular systolic function with an ejection fraction of 55%, poorly visualized calcified AV with moderate to severe stenosis by gradients (peak and mean gradients of 59.2 mmHg and 33.5 mmHg, respectively), and moderate aortic insufficiency. A TEE was performed due to poor TTE imaging windows, which showed a severely calcified bicuspid aortic valve (BAV) with moderate to severe stenosis and mild ascending aortic dilatation of 40 mm. Cardiac catheterization revealed triple vessel coronary artery disease with only one bypassable target to the posterior descending artery (PDA). Computed tomography (CT) angiography of the aorta was performed due to the patient’s history of TS, hypertension, and bicuspid AS to rule out aortopathy. It also demonstrated fusiform dilatation of the ascending aorta with a maximal diameter of 41 mm, calcified AV, and left-sided aortic arch with a retroesophageal right subclavian artery originating from a diverticulum of Kommerell measuring 2.2 cm.

Secondary to the severity of the patient’s symptoms and the imaging results, the patient was transferred to our institution and scheduled for AV replacement in addition to replacement of the ascending aorta and single-vessel coronary artery bypass grafting (CABG).

**Figure 1:**
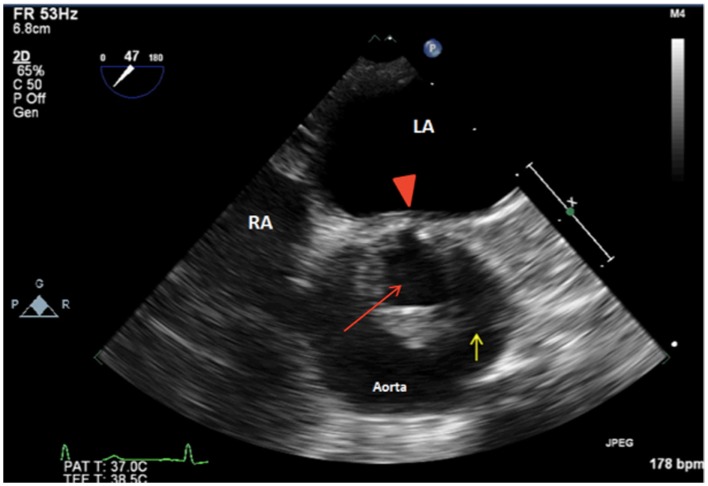
**2D TEE midesophageal AV short-axis view showing a unicuspid aortic valve with a single posterior commissure (arrowhead), raphe (yellow arrow), and an eccentric orifice in systole (red arrow). LA, left atrium; RA, right atrium**.

**Figure 2:**
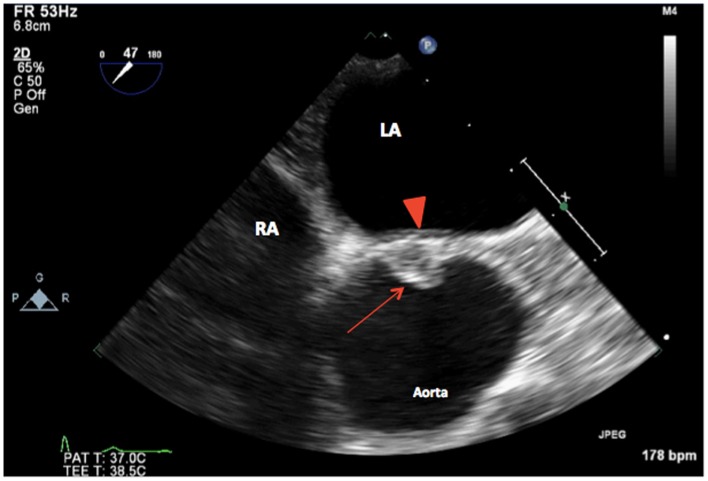
**2D TEE midesophageal AV short-axis view showing a unicuspid aortic valve (arrow) with a single posterior commissure in diastole (arrowhead). LA, left atrium; RA, right atrium**.

**Figure 3:**
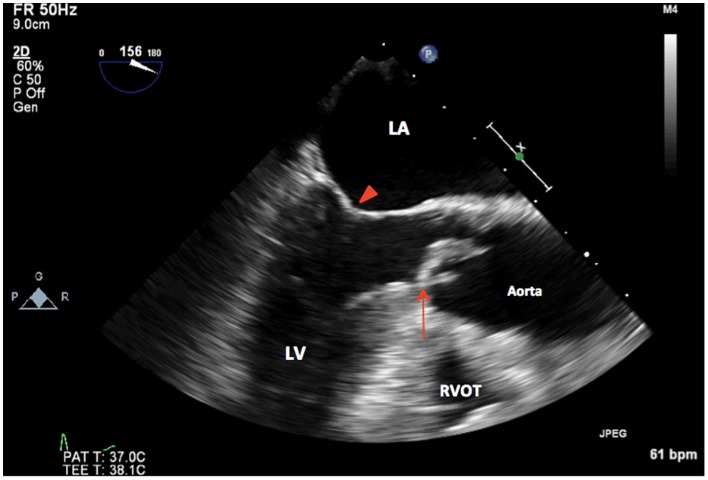
**2D TEE midesophageal long-axis view showing thickened, calcified unicuspid aortic valve in systole (arrow). LA, left atrium; LV, left ventricle, mitral valve (arrowhead); RVOT, right ventricular outflow tract**.

**Figure 4:**
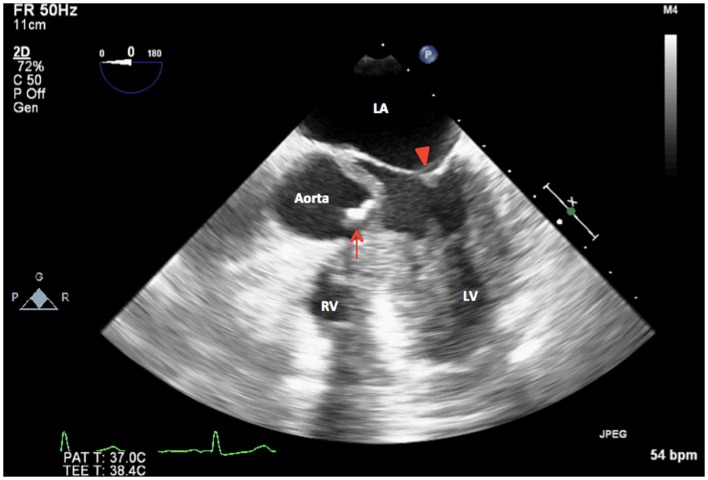
**2D TEE midesophageal modified five-chamber view showing severely calcified and restricted unicuspid aortic valve in systole (arrow). LA, left atrium; LV, left ventricle, mitral valve (arrowhead); RV, right ventricle**.

**Figure 5:**
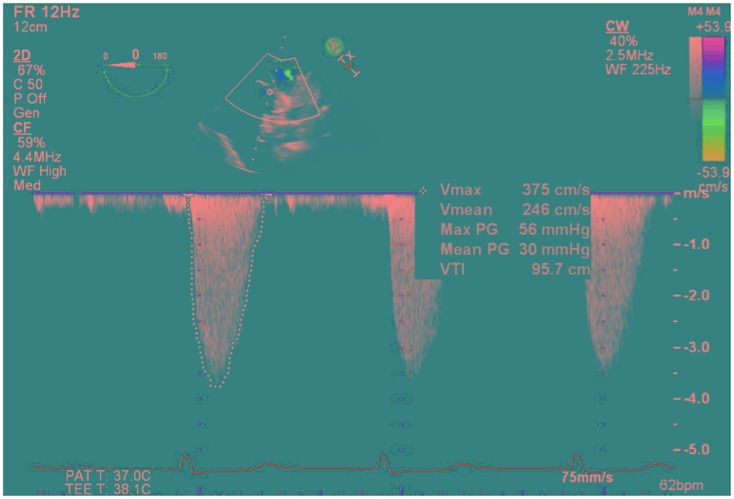
**Deep transgastric view continuous-wave Doppler through the unicuspid aortic valve showing gradients across the valve (peak/mean gradients = 56/30 mmHg, Peak velocity of 3.75 m/s, VTI = 96 cm)**.

Intraoperative TEE assessment showed a moderate to severely calcified UAV with an eccentric orifice and a single posterior commissure (Figures 1–4). The severity of AS was quantified by transvalvular gradients (peak and mean gradients of 56 mmHg and 30 mmHg, respectively) and a peak velocity of 3.75 m/s (Figure 5). The ascending aorta was also dilated and measured 41 mm at the level of the right pulmonary artery.

Direct surgical inspection of the AV after aortotomy confirmed the presence of a UAV with a single raphe and a single posterior commissure. The patient underwent an uneventful AV replacement with a 19 mm Carpentier–Edwards Perimount Magna valve (Edwards Lifesciences Corporation) and a single-vessel CABG to the PDA with a saphenous vein graft. Additionally, ascending aorta replacement with a 28-mm Hemashield graft was also performed. The diverticulum of Kommerell was not resected due to the absence of respiratory and gastrointestinal symptoms in the patient. The patient was subsequently transferred to the cardiovascular intensive care unit, where she was extubated 7 hours later and discharged home on postoperative day 6.

## Discussion

To our knowledge, this is the second reported case of concomitant unicuspid AS and ascending aortic dilatation in a patient with TS (8). Preoperative TTE and TEE misdiagnosed the AV morphology as bicuspid likely due to extensive leaflet calcification and the presence of a raphe; however, the correct diagnosis of UAV was made only by intraoperative TEE. CT angiography was performed to rule out aortopathy, and failed to delineate AV morphology as expected.

Electrocardiographically (EKG) gated cardiac multidetector CT, provides the best assessment of both native and prosthetic AV leaflet morphology and function, and would have clearly diagnosed unicuspid AS (1, 9). In addition, it also provides excellent imaging of ascending aortic pathology, eliminating motion artifacts that can mimic aortic dissection on CT without EKG gating (1). The main limitation of EKG gated cardiac multidetector CT is its failure to assess the hemodynamic severity of valvular disease (9). In our opinion, this limitation is minimal compared to the morphological data that it provides. Of note, EKG gated cardiac multidetector CT was not performed in our case because the diagnosis of severe bicuspid AS, and aortopathy had been clearly determined by echocardiography and CT angiography. It was felt that the additional data provided would not have altered surgical planning and patient outcomes.

A UAV is a rare congenital cardiac anomaly with an estimated incidence of 0.02% in the general population and 4–6% in patients presenting for isolated AV replacement (3, 10). UAV results when two of the commissures fail to develop between the three cusps that normally form the AV during valvulogenesis (11). Two forms of UAV have been described, which consist of unicommissural and acommissural valves. The former is the most common and creates a larger effective orifice area that allows better blood flow into adulthood. The latter is characterized by a central orifice and earlier clinical presentation from premature valvular stenosis during childhood (3, 12).

Aortic valve malformations are present in 10–30% of patients with TS, with 10–18% being bicuspid valves. However, the incidence of UAV in this population remains unknown. These AV anomalies are associated with an increased risk of endocarditis, aortic root and ascending aortic dilatation, aortic aneurysms, and aortic dissections in TS patients (7, 13). The mortality rate in TS patients from aortic dissections is 8% and should be investigated and treated with urgency. Risk factors for aortic dilatation include hypertension, coarctation of the aorta, and AV anomalies (14, 15).

Extensive imaging of the aorta with CT or magnetic resonance imaging to rule out aortopathy, should accompany the diagnosis of AS in this patient population due to the high association with concomitant aortic dilatation and aortopathy, such as diverticulum of Kommerell.

Echocardiography remains the cornerstone for the diagnosis of valvular abnormalities, including unicuspid AS (16). However, TTE has been shown to poorly visualize or define AV morphology in 5–40% of patients (7, 14). A sensitivity of 27% and specificity of 50% have been reported in the TTE diagnosis of UAV secondary to extensive diffuse leaflet calcification obscuring leaflet morphology, compared to 75 and 86%, respectively, by TEE (15). Echocardiographic characteristics of UAV include the visualization of a single AV leaflet with an eccentric orifice and single commissural attachment zone in unicommissural valves, whereas acommissural valves have a central orifice without a commissural attachment. Misdiagnosis of a UAV for bicuspid or tricuspid AV can result from visualization of a raphe or calcifications mimicking a raphe, which can resemble true commissures in diastole (3, 11, 15).

## Conclusion

This case report highlights the importance of detailed intraoperative TEE assessment of AV morphology in patients with AS to help diagnose rare malformations, such as UAV, rather than relying completely on preoperative imaging results. The surgical technique and outcome did not change in this case because the patient had a preoperative CTA showing significant aortopathy. Patients with TS and congenital AV stenosis, including unicuspid AS on echocardiography, should have a detailed assessment of the ascending and descending aorta to ensure absence of aortopathy before proceeding with surgery.

## Conflict of Interest Statement

The authors declare that the research was conducted in the absence of any commercial or financial relationships that could be construed as a potential conflict of interest.
